# Assessment of groundwater hydrochemistry, water quality, and health risk in Hainan Island, China

**DOI:** 10.1038/s41598-023-36621-3

**Published:** 2023-07-26

**Authors:** Qingqin Hou, Yujie Pan, Min Zeng, Simiao Wang, Huanhuan Shi, Changsheng Huang, Hongxia Peng

**Affiliations:** 1 School of Geography and Information Engineering, China University of Geosciences, Wuhan, 430074 China; 2 The second Institute of Resources and Environment Investigation of Henan Province, Henan, 471023 China; 3 College of Environmental Sciences and Engineering, Peking University, Beijing, 100000 China; 4 Wuhan Center of Geological Survey of China Geological Survey, Wuhan, 430000 China; 5 School of Mechanical Engineering and Automation, Northeastern University, Liaoning, 110819 China; 6 School of Environmental Studies, China University of Geosciences, Wuhan, 430074 China; 7 School of Geography and Information Engineering, China University of Geosciences, No. 68, Jincheng Street, East Lake New Technology Development Zone, Wuhan, 430078 Hubei China; 8 Hubei Key Laboratory of Regional Ecology and Environmental Change, China University of Geosciences, Wuhan, China

**Keywords:** Environmental sciences, Environmental chemistry, Environmental impact

## Abstract

Groundwater is an important source of water for human sustenance. The determination of groundwater quality at island sites is an urgent priority in China, but there are lacking systematic reports relating to them. Here, 63 groups of groundwater samples were collected and analyzed of Hainan Island. The groundwater in the study area is weakly alkaline, mainly comprising hard and soft freshwater. The predominant anions and cations are HCO_3_^−^, and Ca^2+^ and Na^+^, respectively, and the main water chemistry types are HCO_3_–Cl–Na and HCO_3_–Cl–Na–Ca. The chemical evolution of groundwater is mainly affected by water–rock interactions, cation exchange, and human activity. The groundwater is mostly of high quality and, in most areas, is suitable for drinking and irrigation. Contrastingly, the water quality in the west of the island is relatively poor. The spatial distribution of the risk coefficient (HQ) is consistent with the spatial variation in the NO_3_^−^ concentrations in the groundwater. Notably, there are unacceptable health risks for different groups of people, with infants having the greatest level of impact, followed by children, teenagers, and adults. This study provides a valuable reference for the development and utilization of groundwater resources, as well as the improvement of aquatic ecological conditions on Hainan Island and other island areas worldwide.

## Introduction

Groundwater is an important source of water for consumption, irrigation, and industrial use^1–4^. However, the improvement in people’s living standards and the degree of industrialization has resulted in a perpetual increase in the demand for water resources^5–7^, consequently leading to the over-exploitation of global groundwater resources, deterioration in water quality, and worsening of water security problems^8–11^. In particularly, groundwater resources face severe challenges, especially in island areas with more fragile natural ecosystems^12–14^. Most of the island areas have low rainfall, large evaporation, serious water and soil loss, and relatively lack of surface water resources. Therefore, the exploitation and utilization of groundwater is extremely important for the production and life of residents. The island groundwater system is an independent circulating system with limited supply sources. If water quality pollution is caused by natural factors and human activities, it may cause irreversible losses.

The chemical composition of groundwater is the result of its long-term interactions with the surrounding environment^15^. During groundwater formation and migration, physical and chemical interactions occur with the surrounding media, which affect the chemical composition of the water^16,17^. Simultaneously, groundwater is increasingly being affected by human factors^18,19^. Harmful substances produced by humans may enter groundwater, and the resulting pollution, which is spread via groundwater flows, can penetrate deep into the ground. In particular, NO_3_^−^ produced by industrial production, agricultural activities and domestic sewage will enter shallow or even deep groundwater with rainwater or surface water, affecting the water quality and hydrochemical evolution process^20^. At present, nitrate pollution has become one of the major pollutants in the global groundwater and has caused potential health risks to residents^21,22^. The water quality index (WQI) simplifies the complex water quality index into a single value, which can be used to evaluate the water quality more intuitively and effectively^23^. At present, it has been widely used in the evaluation of drinking and irrigation water, and even innovatively used by some scholars to evaluate the suitability for industrial use. Nsabimana et al. and Li et al. used a new industrial water quality index (Ind WQI) model to determine the overall industrial water quality^24^. According to the determination of the main influencing factors in water quality assessments, the health risk assessment model and the assessment standard provided by the U.S. Environmental Protection Agency (USEPA) can be used to further assess risks to human health^25^.

Hainan Island is China’s second largest island, with one of the largest special economic zones and free trade ports, serving as the country’s major development strategy. Due to its location advantages and policy support, Hainan Island has undergone rapid modernization and the establishment of numerous industrial and agricultural parks. This rapid economic development has also increased pressure on residential water supplies and caused a series of ecological and environmental issues, including pollution, soil salinization, and seawater intrusion^26,27^. However, current research on groundwater on Hainan Island remains focused on hydro-chemical exploration, with a lack of comprehensive research on water quality evaluation and human health risk.

To address this research gap, the main goals of this study were to (1) analyze the groundwater hydro-chemical characteristics and the main controlling factors on Hainan Island; (2) evaluate the suitability of groundwater for drinking and irrigation; and (3) assess the risk of the nitrates in groundwater to human health. Our findings will help to better understand the chemical characteristics and quality of groundwater in this area of China, and provide a reference for the development and utilization of groundwater resources and the improvement of the aquatic ecological environment.

## Materials and methods

### Study area

**Figure 1:**
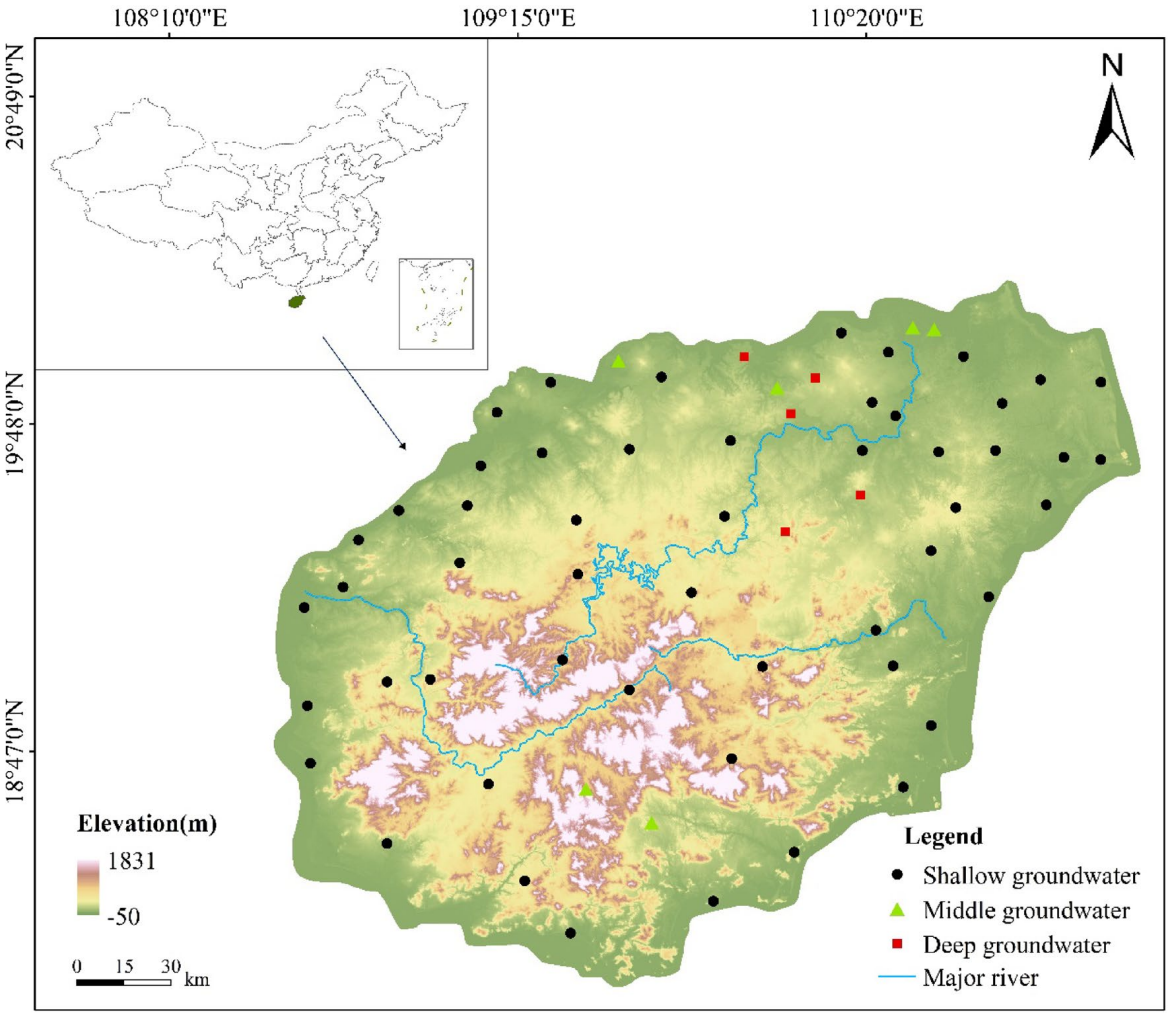
Groundwater sampling points on Hainan Island, China. The map was created using ArcGIS 10.8 (https://www.esri.com/software/ArcGIS).

Hainan Island is located at the southernmost tip of China (18° 10′–20° 10′ N and 108° 37′–111° 03′ E; Fig. 1). Hainan is surrounded by the sea, located in the tropics and subtropics, and receives abundant precipitation; however, the problems of regional and seasonal precipitation are prominent. High temperatures throughout the year drive strong evaporation. The long-term annual average rainfall in Hainan is 1750 mm, but it is unevenly distributed. The central and northeastern parts of the island receive more rain than the southwest regions. Hainan Island is low and flat, with raised topography in the center of the island, which limits the options for surface water storage. As China’s largest provincial-level special economic zone, the island has a growing population density and is experiencing rapid industrial development, which has resulted in high water demand, leading to a scarcity of water resources. At the end of the 1970s, the amount of groundwater exploitation in Hainan Island was only 290 million m^3^/a. After the construction of Hainan Island as a province, the amount of groundwater exploitation gradually increased due to economic development and technological progress. In 2004, the amount of groundwater exploitation increased to 515 million m^3^/a. Since then, due to the introduction of the national underground water pipe control policy and the enhancement of the development and utilization capacity of surface water, the mining capacity has gradually decreased, and the water supply of underground water in 2015 is still 274 million m^3^/a.

According to the type of water-bearing medium and occurrence conditions, the groundwater in Hainan Island can be divided into five types: bedrock fissure water, pore-confined water of loose and semi-consolidated rock, volcanic rock pore fissure water, carbonate rock fissure karst water, and loose rock pore phreatic water. The main aquifer strata are Quaternary, Neogene, Cretaceous and Triassic. The groundwater in the area is mainly supplied by atmospheric rainfall, and some sections are supplied by surface water. Its runoff generally follows a complete hydrogeological unit, its flow direction is perpendicular to the contour line, and flows from high to low. With Wuzhi Mountain, Limu Mountain, Diaoluo Mountain, etc. as the core, the groundwater runoff flows around and radiates, and discharges along the coast. Generally, it is discharged to rivers, lakes, or discharged to the ground in the form of springs and scattered wetlands. Artificial drainage of groundwater has also become an important form of discharge.

### Sample collection and testing

A total of 63 groups of groundwater samples were collected from civilian wells, pump wells and springs, with the sampling well depth between 3.5 and 340 m. According to the sampling depth, it is divided into shallow groundwater (0–20-m deep), middle groundwater (20–50-m deep) and deep groundwater (> 50-m deep), which are respectively from phreatic water, middle confined water and deep confined water. The water temperature, pH, conductivity, dissolved oxygen, total dissolved solids, and redox potential were measured on-site using a portable water-quality analyzer (HQ-40d, HACH, America). The collected water samples were filtered using 0.45-μm microporous filter membranes and then packed in 500-mL polyethylene plastic sample bottles that had been rinsed with deionized water at least three times. The samples used for the determination of cations were acidified to pH < 2 with approximately 3 mL of 65% HNO_3_. The samples for anion detection, without any modification, were sealed and stored in a refrigerator at 4 °C. The processed samples were tested by the Changsha Mineral Resources Supervision and Testing Center, Ministry of Land and Resources. The contents of K^+^, Na^+^, Ca^2+^, and Mg^2+^ were determined using a plasma-generation spectrometer (ICP-MS; 7700X, Agilent Technologies, Japan); the contents of SO_4_^2−^, Cl^−^, and NO_3_^−^ were measured by ion chromatography (ICS-1100, Thermo Scientific, America); and the content of HCO_3_^−^ was determined by titration. The detection limit of each ion was 0.01 mg/L and the measurement error was less than 0.1%. After all the analysis procedures were completed, the charge balance error (CBE) was calculated by the following formula:

1 $${\text{CBE = 100\% }} \times \left( {\frac{{{\text{meq/L}},{\text{cations}} - {\text{meq/L}},{\text{anions}}}}{{{\text{meq/L}},{\text{cations}} + {\text{meq/L}},{\text{anions}}}}} \right).$$

In the study, the average value of *CBE* is less than 5%, indicating that the analysis result is reasonable.

### Data processing

#### Water quality index (WQI)

The WQI is an effective tool for appraising the overall quality of groundwater^28^, and is calculated as:

2 $$W_{i} = \frac{{w_{i} }}{{\sum {w_{i} } }},$$

3 $$WQI = \sum {\left( {W_{i} \times \left( {\frac{{C_{i} }}{{S_{i} }}} \right) \times 100} \right)} ,$$

4 $$EW_{{\text{i}}} = \frac{{W_{i} \times \left( {\frac{{C_{i} }}{{S_{i} }}} \right)}}{WQI} \times 100,$$

where *i* represents the sample number; ${W}_{i}$ and ${w}_{i}$ are the relative weight and weight of each index, respectively (Table S1); ${C}_{i}$ and ${S}_{i}$ are the measured concentration and permissible value of each index, respectively; and *EW*_i_ is the effective weight of each index. *WQI* values can be categorized as non-drinkable (*WQI* ≥ 300), very poor (200 ≤ *WQI* < 300), poor (100 ≤ *WQI* < 200), good (50 ≤ *WQI* < 100), and excellent (*WQI* < 50).

#### Irrigation water quality

Understanding the properties of groundwater is essential in areas where it is used as a source of irrigation. For example, an excessive salt content can result in sodium and salinity hazards^29,30^. In this study, the irrigation water quality was assessed based on the sodium adsorption ratio (SAR), soluble sodium percentage (% Na), and residual sodium carbonate (RSC), as follows:

5 $$SAR = \frac{{{\text{Na}}^{ + } }}{{\frac{{\sqrt {{\text{Ca}}^{{{2} + }} + {\text{Mg}}^{{{2} + }} } }}{{2}}}},$$

6 $$\% {\text{Na}} = \frac{{{\text{Na}}^{ + } + {\text{K}}^{ + } }}{{{\text{Na}}^{ + } + {\text{K}}^{ + } + {\text{Ca}}^{{{2} + }} + {\text{Mg}}^{{{2} + }} }},$$

7 $$RSC = \left( {{\text{CO}}_{{3}}^{{{2} - }} + {\text{HCO}}_{{3}}^{ - } } \right) - \left( {{\text{Ca}}^{{{2} + }} + {\text{Mg}}^{{{2} + }} } \right),$$

where all the ionic concentrations of the respective ions are expressed in milliequivalents per liter (meq/L).

#### Health risk assessment

The health risk assessment models and standards provided by the USEPA have been widely used to quantitatively assess potential hazards. Previous studies suggested that the oral ingestion of groundwater pollutants is more harmful to human health than inhalation and skin contact, and several factors that are human health risks induced by skin-contact pollutants are relatively uncertain. Therefore, we assessed the threat of nitrate pollution to human health through drinking using^28,29^:

8 $$HQ = \frac{E}{{R{\text{f}}D}},$$

9 $$E = \frac{C \times IR \times EF \times ED}{{BW \times AT}},$$

where *HQ* is the non-carcinogenic risk coefficient; *E* and *RfD* are the exposure dose and reference dose, respectively; *C* is the measured nitrate concentration; *IR* is the daily water consumption; *EF* is the exposure frequency; *ED* is the exposure duration; *BW* is the average body weight; and *AT* is the average lifetime. Table S2 shows the parameters of the health risk assessment model used to assess the risk from groundwater nitrates on Hainan Island^31–34^.

Monte Carlo simulation is a random number based calculation method used to simulate probability distribution functions, suitable for simulating highly complex phenomena that traditional analytical methods are difficult to solve. Its basic idea is to simulate a set of random variables that conform to the probability distribution function through random sampling, and perform numerical calculations or statistical analysis based on these random variables to obtain statistical quantities or numerical results. This method can to some extent reduce the impact of exposure parameter uncertainty in health risk models. The concentration of nitrate (C), adult weight (BW), and ingestion rate (IR) were considered as variable parameters, and distribution functions were shown in Table S3^21^. Monte Carlo simulation was performed using Crystal Ball 11.1.2.4 and iterated 10,000 times to ensure the robustness of the study.

## Results and discussion

### Groundwater chemical characteristics

#### Descriptive statistics

The pH of groundwater on Hainan Island ranged from 5.11 to 9.37, with an average of 7.47, indicating weak alkalinity (Table 1). According to the TDS content, the underground water can be divided into fresh water (TDS < 1000 mg/L) and brackish water (TDS > 1000 mg/L). According to the TH content, the underground water can be divided into soft water (TH < 150 mg/L) and hard water (TH > 150 mg/L). The range and mean value of TDS were 30.95–1077.30 and 287.41 mg/L, respectively, with only one water sample exceeding 1000 mg/L. The range and mean TH were 5.31–495.72 and 121.91 mg/L, respectively, indicating that the island’s groundwater is a combination of both hard and soft freshwater.

**Table 1:** Mass concentration statistics of the main hydro-chemical indexes.

	Units	Mean	SD	CV	Min	Median	Max
TDS	mg/L	287.41	216.13	0.75	30.95	235.02	1077.3
pH	–	7.47	1.03	0.14	5.11	7.53	9.37
TH	mg/L	121.91	103.77	0.85	5.31	94.50	495.72
Ca^2+^	mg/L	30.25	26.41	0.87	0.71	22.19	121.4
Mg^2+^	mg/L	11.28	13.3	1.18	0.69	7.29	80.88
K^+^	mg/L	13.16	21.13	1.61	0.9	4.69	130.4
Na^+^	mg/L	30.3	29.6	0.98	2.82	21.51	166.2
Cl^-^	mg/L	43.88	55.99	1.28	0.87	27.14	351.24
SO_4_^2-^	mg/L	29.39	42.12	1.43	0.13	10.81	203.78
HCO_3_^-^	mg/L	111.29	98.49	0.89	4.25	70.16	365.47
NO_3_^-^	mg/L	38.92	51.52	1.32	0	15	226.26
%Na	–	42.27	17.03	0.4	10.28	41.91	82.36
SAR	–	1.23	0.76	0.62	0.18	1.01	3.60
RSC	meq/L	− 0.63	1.07	–	− 4.61	− 0.36	0.99

*Min* minimum, *Max* maximum, *SD* standard deviation, *CV* coefficient variation.

The relative abundances of the major cations in the sampled groundwater were in the order of Ca^2+^ > Na^+^ > Mg^2+^ > K^+^, whereas those of the anions were in the order of HCO_3_^−^ > NO_3_^−^ > Cl^−^ > SO_4_^2−^. The dominant cation was HCO_3_^−^, which accounted for 42.42% of the total anion concentration, whereas the dominant cations were Ca^2+^ and Na^+^, which accounted for 36.83% and 32.07% of the total cation concentration, respectively. The coefficient of variation of the main ions in the groundwater ranged from 0.14 to 1.61, with the values of Mg^2+^, K^+^, Cl^−^, SO_4_^2−^, and NO_3_^−^ exceeding 1. This indicates that the spatial distribution of these ions was significantly different, with a high degree of local enrichment.

The nitrate concentration in the groundwater samples was 0–226.26 mg/L, with an average of 38.92 mg/L. Based on these results, 41.27% of the samples exceeded the class III value of 20 mg/L, as specified in China’s groundwater quality standard (2017), which was mainly attributable to the discharge of domestic sewage and industrial and agricultural activities.

#### Hydro-chemical classification of groundwater

Groundwater chemistry is closely correlated with water quality^35^. Piper diagrams are often used to examine the general chemical characteristics and types of groundwater. The Piper diagrams for Hainan Island showed that the predominant cations comprised Ca^2+^ and Na^+^ + K^+^ terminal members, and the predominant anions comprised HCO_3_^−^ terminal members, which may be mainly related to the rich rainfall and the dissolution of carbonate minerals in the study area (Supplementary Fig. S1). According to the Schukalev classification, the hydro-chemical types of the regional groundwater were, therefore, HCO_3_–Cl–Na and HCO_3_–Cl–Na–Ca. In the Piper diagram, the groundwater sample points in the study area are relatively scattered and there are many types of hydrochemical types, indicating that the groundwater chemical characteristics vary greatly and may be affected by natural and human factors.

### Factors controlling groundwater chemistry

#### Natural factors

**Figure 2:**
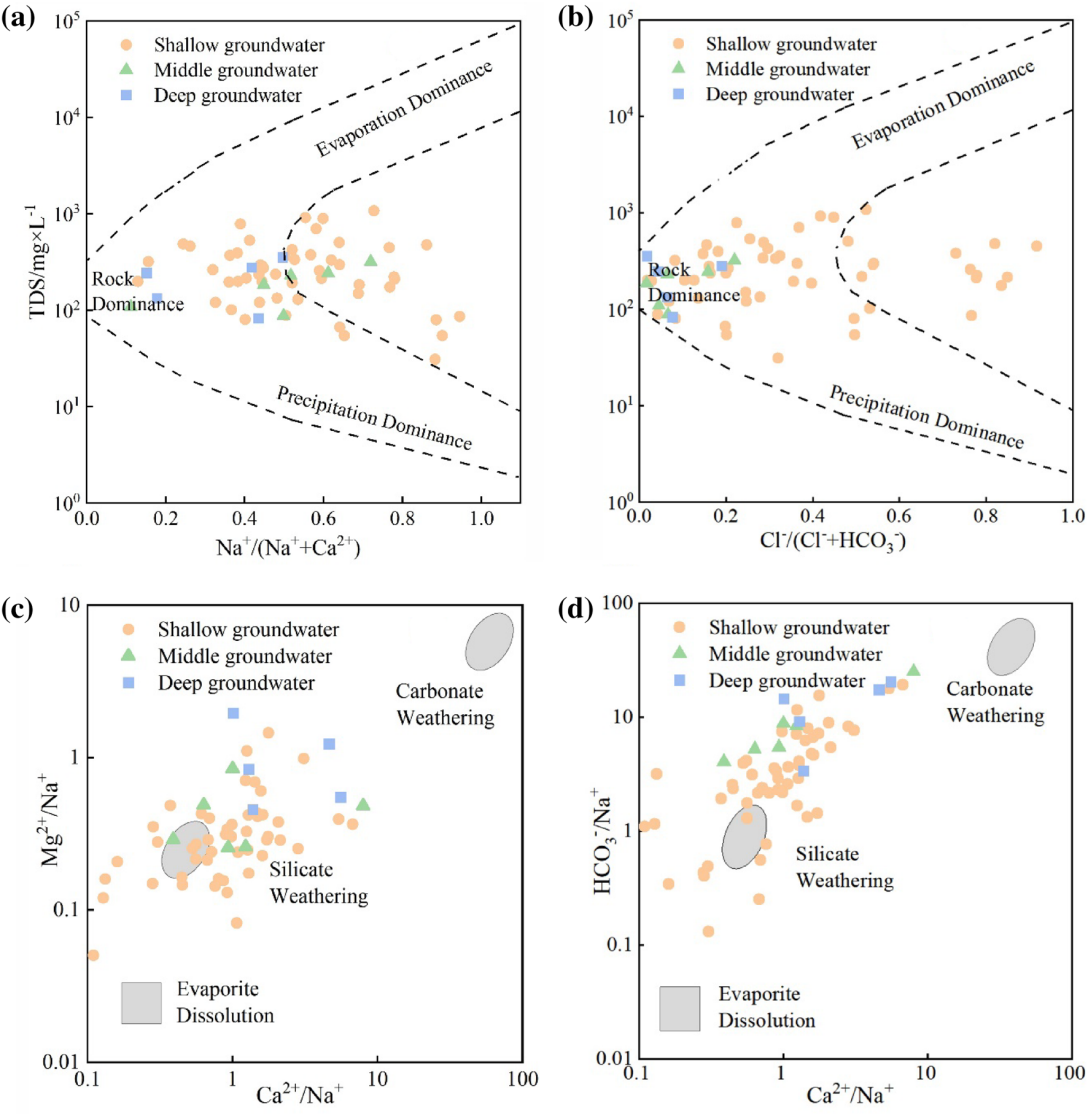
Gibbs diagrams of groundwater hydro-chemistry for (**a**) total dissolved solids (TDS) versus Na^+^/(Ca^2+^ + Na^+^); (**b**) TDS versus Cl^−^/(Cl^−^ + HCO_3_^−^); (**c**) Mg^2+^/Na^+^ versus Ca^2+^/Na^+^; and (**d**) HCO_3_^−^/Na^+^ versus Ca^2+^/Na^+^.

Gibbs diagrams, which divide formation mechanisms into natural factors, including precipitation, rock weathering processes, and evaporation, are widely employed to explore groundwater formation mechanisms. These diagrams have, indeed, been applied by many scholars to assess groundwater evolution^36^. The groundwater samples from Hainan Island were mainly distributed in the “rock dominance” area, with a few falling in the “precipitation dominance” area (Fig. 2a,b). This suggests that rock weathering processes dominated the groundwater chemistry in the study area. Precipitation also had some influence on groundwater chemistry, whereas evaporation (and crystallization) appeared to have little influence. Some of the shallow groundwater sample points fell outside of the model block diagram, indicating a stronger influence of human activities.

The effect of rock weathering on the hydro-chemical evolution of groundwater can be further explored using endmember diagrams. Weathering sources can be divided into carbonate weathering^37^, silicate weathering, and evaporite dissolution based on the ratios of Mg^2+^/Na^+^, Ca^2+^/Na^+^, and HCO_3_^−^/Na^+^. The groundwater samples from the study area were mainly plotted between the silicate and carbonate weathering endmembers, with only a few samples plotted between the silicate weathering and evaporite dissolution endmembers (Fig. 2c,d). In contrast to the shallow groundwater samples, the middle and deep groundwater samples tended toward the carbonate mineral endmembers. This implies that the weathering of silicate and carbonate minerals plays a major role in the evolution of groundwater on the island, with a weaker contribution from evaporite dissolution.

**Figure 3:**
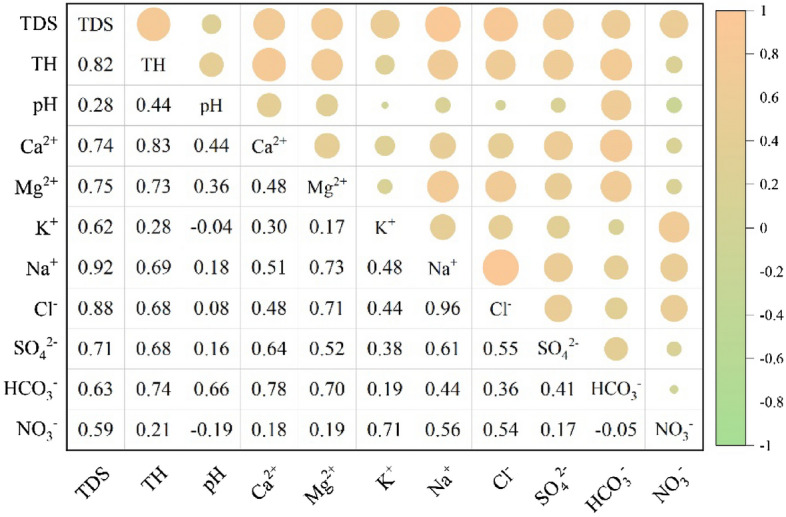
Correlation matrix between water chemistry variables. p < 0.10, **p < 0.05, ***p < 0.01.

Figure 3 shows the Pearson correlation coefficient matrix between the measured groundwater chemical parameters. A strong significant, positive correlation can be observed between Na^+^ and Cl^−^ (r = 0.92), indicating that the two ions had similar sources. The ratio of Na^+^ and Cl^−^ can also indicate the sources of Na^+^ and K^+^ in groundwater^38^. Most of the Hainan samples were plotted to the left of the 1:1 equivalent line (Supplementary Fig. S2a), indicating that the excess Na^+^ and K^+^ in the groundwater may have originated from the weathering of silicate rocks or cation exchange.

Ca^2+^ and Mg^2+^ were significantly, positively correlated with HCO_3_^−^ (r = 0.78 and r = 0.70, respectively), indicating a common source. The sources of Ca^2+^ and Mg^2+^ can be determined by (Ca^2+^ + Mg^2+^)/HCO_3_^−^: > 1, indicating that the dissolution of carbonate rocks is likely dominant, and < 1, indicating that the dissolution of silicate and evaporite rocks are considered dominant^39^. For Hainan Island, most of the middle and deep groundwater samples were plotted to the lower right of the 1:1 line (Supplementary Fig. S2b), indicating that the Ca^2+^ and Mg^2+^ in these waters were mainly derived from the dissolution of silicates and evaporites. In contrast, the ratios of 71.15% of the shallow groundwater samples were > 1, the dominance of the dissolution of carbonate rocks.

The ratio of Cl^−^ + SO_4_^2−^ and HCO_3_^−^ can also be used as an index to distinguish the relative contributions of the weathering of different types of rocks. Both the middle and deep groundwater samples were plotted to the upper left in Supplementary Fig. S2c, indicating that the dissolved ions in these waters were mainly affected by evaporite rocks. In comparison, the shallow groundwater samples were distributed on both sides of the 1:1 line, indicating inputs from both evaporite and carbonate rocks.

Through their long-term interaction, the negative charges carried by rock surfaces can adsorb cations from and release cations to groundwater, i.e., alternate cation adsorption can occur. The possibility of alternate cation adsorption can be determined by the relationship (Mg^2+^ + Ca^2+^–SO_4_^2–^HCO_3_^−^)/(Na^+^ + K^+^–Cl^−^), whereby ratios closer to − 1 indicate cation exchange^32^. Most of the shallow, middle, and deep groundwater samples from Hainan Island were plotted around the − 1 ratio line (Supplementary Fig. S2d), indicating alternate cation adsorption.

The direction and intensity of alternate cation adsorption can be further expressed using the Chloron–Alkaline Index (CAI). In this case, when the Ca^2+^ and Mg^2+^ in groundwater are exchanged with Na^+^ and K^+^ in the aquifer, both CAI-I and CAI-II are negative, and when reverse ion exchange occurs, CAI-I and CAI-II are positive^40,41^. For the Hainan Island samples, 88.89% of the CAI values were negative (Supplementary Fig. S2e). This indicates that reverse cation exchange is dominant and likely acts to increase the Na^+^ and K^+^ and decrease the Ca^2+^ and Mg^2+^ concentrations in groundwater. These processes act as an important source of sodium.

#### Anthropogenic inputs

Nitrate has good solubility in water^42^. Therefore, NO_3_^−^ in wastewater, waste gas, and waste produced through human activities can enter shallow and deep groundwater via rainwater or surface water, thereby affecting groundwater quality and water chemistry^43^. The relationship between Cl^−^/Na^+^ and NO_3_^−^/Na^+^ can reflect the influence of groundwater by human activities; the higher the ratio, the stronger the effect of human activities on groundwater chemistry^44^. The ratios of Cl^−^/Na^+^ and NO_3_^−^/Na^+^ were relatively high in the samples from Hainan Island (Supplementary Fig. S2f). Indeed, most of the water samples showed bias towards agricultural activities, with only a few points plotting between carbonate rock and salt rock. This indicates some degree of agricultural pollution on the island. Simultaneously, NO_3_^−^ and K^+^ were strongly correlated (r = 0.71), indicating that agricultural fertilizers, such as potassium fertilizer, that are not fully absorbed by crops enter surface waters or penetrate the groundwater system with irrigation water, resulting in nitrate pollution. The amount of fertilizer applied in Hainan Province is 511,400 tons, including 152,700 tons of nitrogen fertilizer, 40,500 of phosphate fertilizer, 91,100 tons of potassium fertilizer and 227,100 tons of compound fertilizer (Fig. S3). The higher application rate of chemical fertilizer can also support our conjecture.

The groundwater samples with NO_3_^−^ concentrations higher than the class III limit specified by China’s groundwater quality standard (20 mg/L) were mainly obtained from Dongfang City and Danzhou City in the west of the island; Sanya City, Ledong County, and Lingshui County in the south; and Wenchang City and Qionghai City in the northeast and coastal areas (Supplementary Fig. S4a). The high hydrochloride content of the groundwater in these areas may present a certain health risk to the locals; therefore, it cannot be considered suitable as a direct source of drinking water. Given the ongoing development of “tropical agriculture” on Hainan Island, the use of chemical fertilizers needs to be carefully controlled to reduce the impact of agricultural pollution on groundwater quality and avoid damage to the ecological environment.

### Water quality evaluation

#### Adaptability of groundwater for drinking

Groundwater quality assessment is very important for determining regional drinking water safety^45^. In this study, the WQI was used to evaluate the drinking water quality in the study area. The WQI of the groundwater on Hainan Island ranged from 9.96 to 266.10, with an average of 61.37; and 60.32% of the samples were classified as “excellent”, 19.05% as “good”, 14.29% as “medium”, and 4.76% as “poor”. The overall water quality was “good”. The average Ew_i_ for NO_3_^−^ and pH were the highest, at 43.67% and 29.73%, respectively, indicating that these parameters had the greatest impact on the WQI (Table S1).

The groundwater in the study area showed strong spatial variability (Supplementary Fig. S4b). The samples with “good” water quality were mainly distributed in the middle of the island, whereas the samples with “poor” water quality were mainly obtained from the coastal areas of Dongfang City and to the west of Danzhou City. This may partially reflect the west of the island being on the leeward slope of the southeast monsoon. The southeast monsoon is blocked by Wuzhi Mountain; thus, the air in the southwest is relatively dry, with low precipitation. In addition, the rich mineral resources and convenient transport links in the west of the island make its industry develop rapidly, but also affect the quality of groundwater. Thus, a combination of natural and human factors has a major impact on the groundwater quality of the island.

#### Adaptability of groundwater for irrigation

Groundwater is the main water source of water for agricultural irrigation; however, high salinity and sodium content in irrigation water lead to salinization, which reduces soil quality and crop yields^46^. The SAR and %Na values can be used to evaluate these potential effects, and RSC indicates the potential for removing Ca^2+^ and Mg^2+^ from soil solutions. For Hainan Island, the SAR and RSC values were 0.18–3.6 and − 4.16 to 0.99, respectively (Table 1). This indicates that all the groundwater sampling points were suitable for irrigation. However, the %Na values ranged from 10.28 to 82.36, with 14.29% of the samples exceeding the acceptable limit for irrigation by 60.

**Figure 4:**
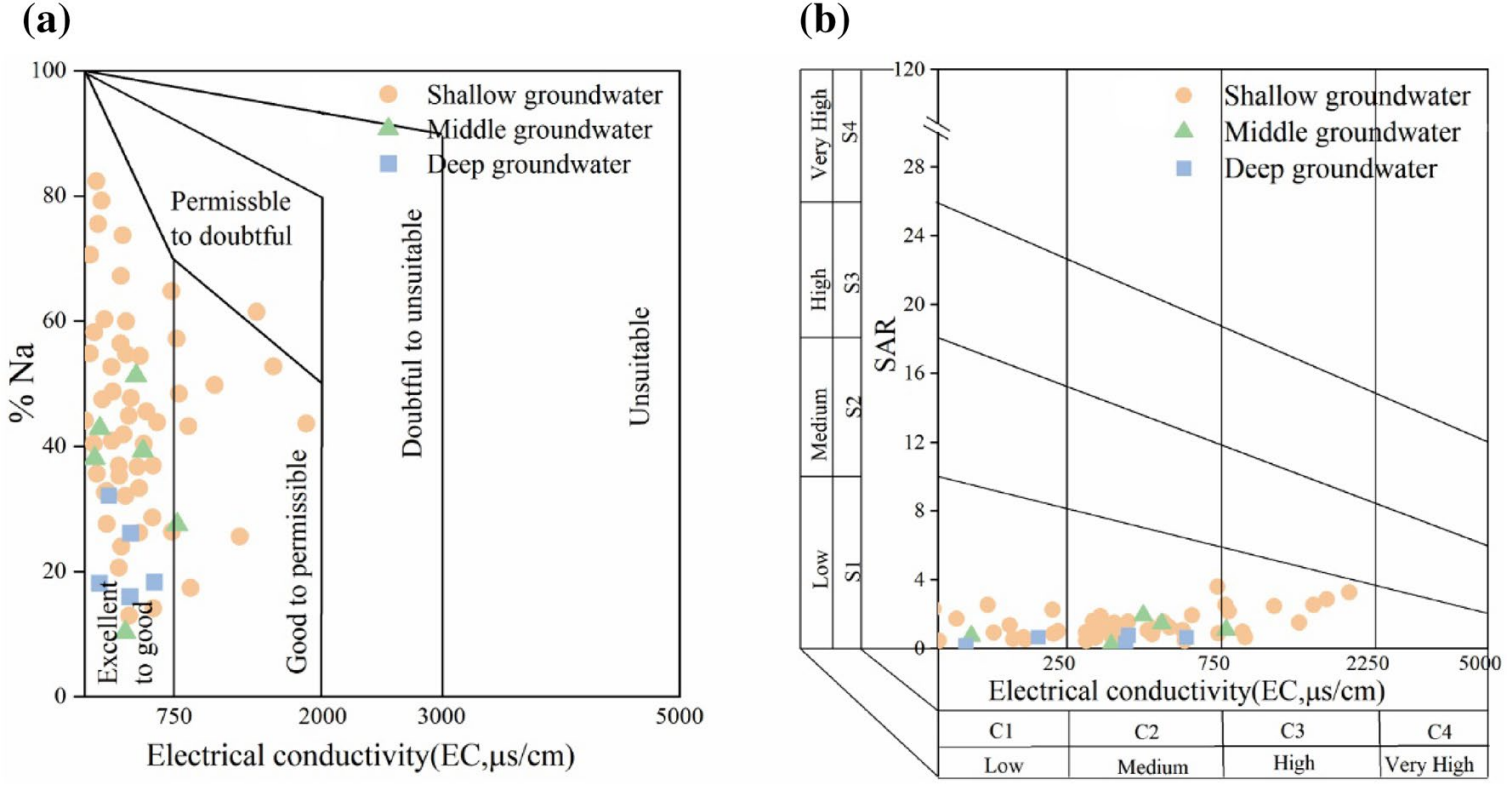
Wilcox (**a**) and USSL (**b**) diagrams for irrigation water quality assessment.

Wilcox and USSL plots can help to evaluate the quality of irrigation water^47,48^. Wilcox plots are divided into five areas—“excellent to good”, “good to permissible”, “permissible to doubtful”, “doubtful to unsuitable”, and “unsuitable”^49^. Most of the Hainan Island samples were distributed in the excellent to permissible categories, with one sample (from Changjiang County) falling into the “permissible to doubtful” category (Supplementary Fig. S4c, Fig. 4a). In the USSL diagram (Fig. 4b), the samples fell into the S1 region, with most distributed in the S1C1 and S1C2 regions and 14.29% in the S1C3 region. All sampling points, except for two, were obtained from Danzhou City, Changjiang County, Dongfang City, and Baisha County in the west of the island (Supplementary Fig. S4d). This may reflect serious seawater intrusion and the widespread distribution of salt fields in the western coastal area of Hainan Island.

Table 2 shows a comparison of irrigation suitability between Hainan Island and other coastal countries. Hainan Island, the Muda Basin in Malaysia^50^, and KwaZulu-Natal in southern Africa^51^ have SAR values below 10, indicating good irrigation suitability. In comparison, 20.83% and 10.34% of northern Algeria^52^ and Dar es Salaam^53^ in Tanzania have SAR values above 10, indicating poor irrigation suitability. Based on %Na, the irrigation suitability of the groundwater on Hainan Island was slightly lower than that of Tamil Nadu in India, but higher than that of a few other regions. The average EC value for the Hainan Island samples was also higher than that of KwaZulu Natal in southern Africa and the Muda Basin in Malaysia, but far lower than those of Tamil Nadu in India, northern Algeria, and Dar es Salaam in Tanzania. Overall, Hainan Island exhibited good groundwater irrigation suitability compared with other regions, with lower salinity and good water quality.

**Table 2:** Comparison of irrigation suitability in Hainan Island and other agricultural areas in China.

Parameters	Range	Percentage of samples (%)				
		Hainan Island	Dar es Salaam, Tanzania	North of Algeria	Muda Basin, Malaysia	KwaZulu-Natal, Africa
SAR	< 10	100	89.575	79.27	100	100
	10–18	–	8.3	20.83	–	–
	18–26	–	2.04	–	–	–
	> 26	–	–	–	–	–
%Na	< 20	11.11	8.30	–	7.44	–
	20–40	33.33	24.3	66.67	33.88	34
	40–60	41.27	36.05	20.83	48.76	55
	60–80	12.70	31.25	12.5	12.40	11
	> 80	1.59	–	–	–	–
EC	Average	444.82	1854.17	2607	340.72	312

### Health risk assessment

When the nitrate content in groundwater exceeds the safe limit, it poses a potential threat to human health^54^. Previous studies have shown that NO_3_^-^ is the main factor affecting water quality. While water quality assessment can indicate whether groundwater is suitable for drinking at the regional scale, it does not reflect the potential health risks caused by local pollution. Therefore, the model recommended by the U.S. Environmental Protection Agency was used to evaluate the impact of groundwater NO_3_^−^ on human health on Hainan Island.

**Figure 5:**
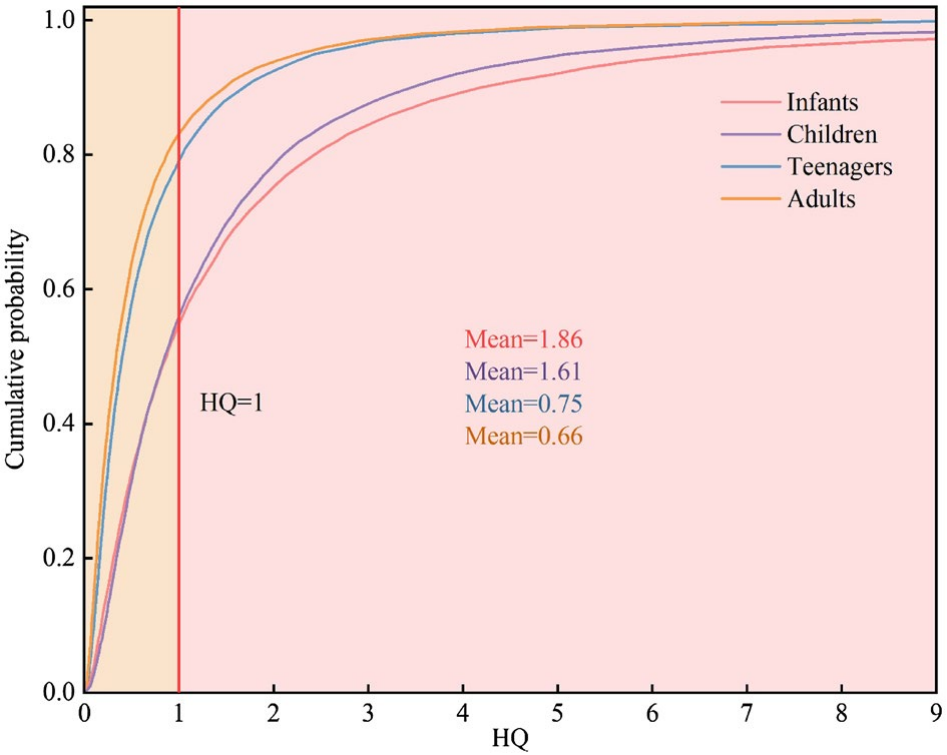
Cumulative probability distribution diagram.

**Figure 6:**
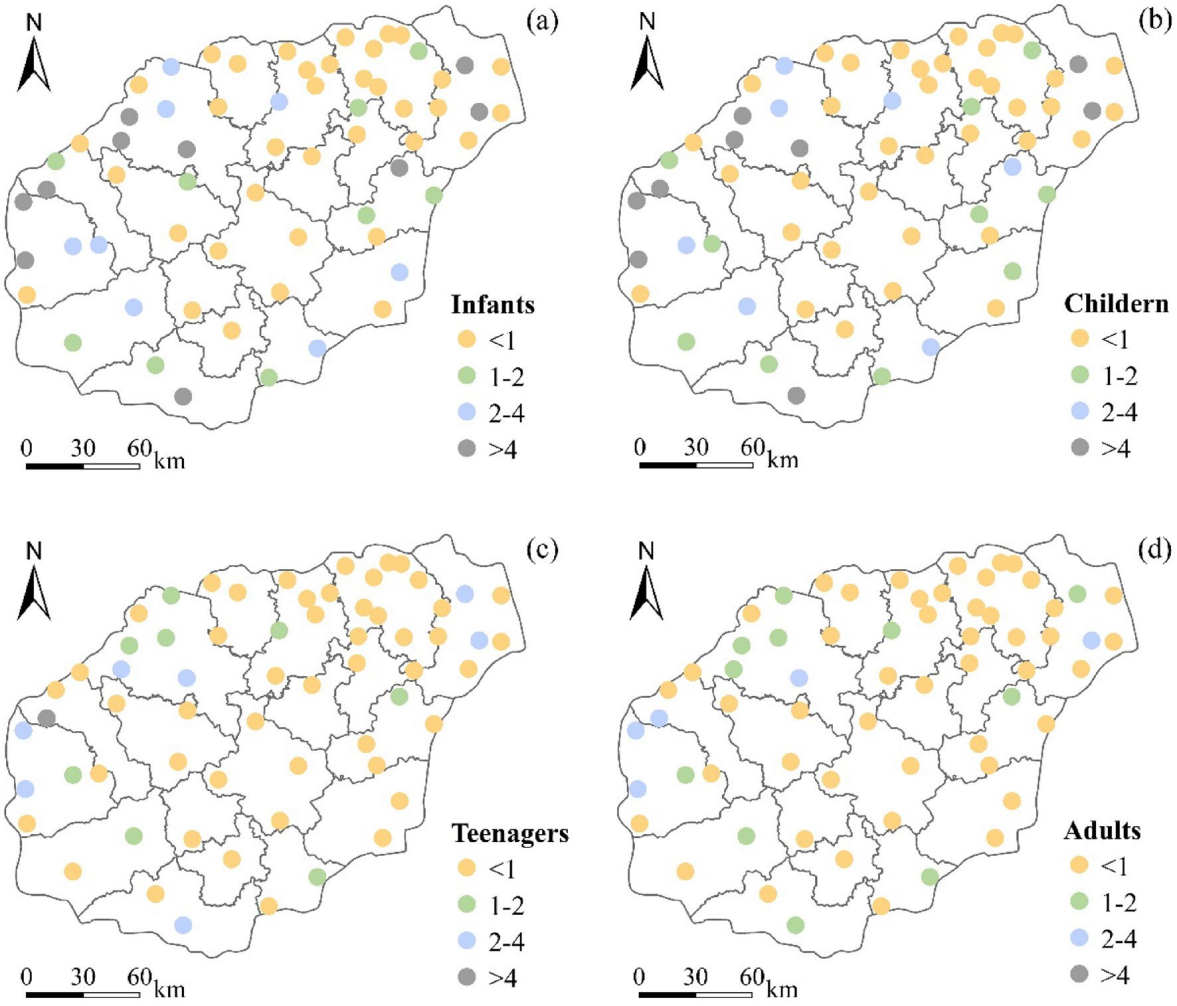
Spatial distribution of the health risks (HQ, hazard quotient) associated with the use of nitrate-contaminated groundwater for drinking by infants (**a**), children (**b**), teenagers (**c**), and adults (**d**). The map was created using ArcGIS 10.8 (https://www.esri.com/software/ArcGIS).

The HQ ranges for infants, children, adolescents, and adults were 0–10.68, 0–9.03, 0–4.16, and 0–3.66, respectively, with the greatest risk for infants and children. The average HQ of infants and children in all groundwater samples was 1.84 and 1.55, respectively, with 46.03% and 44.44% of the samples exceeding the acceptable value of 1. The average HQ for teenagers and adults was 0.72 and 0.63, respectively, with an over standard rate of 25.4%. The uncertainty analysis results of Monte Carlo simulation showed that the mean values of infants, children, adolescents, and adults were 1.85, 1.56, 0.74, and 0.64, respectively, and the probability of exceeding the threshold was 46%, 44%, 21%, and 17%, respectively (Fig. 5). These results are similar to the average value of traditional health risks and the proportion of exceeding acceptable risks, indicating that the results of the two methods are consistent and suitable for human health risk assessment. The NO_3_^−^ health risks reflect the spatial distribution of the NO_3_^−^ concentrations, which were notably high in the west of Danzhou City and Dongfang City, and central Weifang City (Fig. 6).

Numerous studies have shown that groundwater nitrate pollution is mainly caused by human activities, such as the unqualified discharge of domestic sewage and industrial wastewater, agricultural fertilization, and runoff from livestock breeding^55^. With an increase in population and the development of industry and agriculture, nitrate pollution is becoming increasingly common worldwide. This study shows that nitrate pollution in Hainan Island is currently at a medium level, with a lower average value than that in the central and western regions of Jiaokou in northern China^56^, Songnen Plain in northeastern China^4^, and Shandong Peninsula in eastern China, but higher value than that in the North China Plain^57^ and Nanchong in the southwest^58^ (Supplementary Fig. S5). Hainan Island has a higher average concentration of nitrate in its groundwater than that in some regions of other countries, including Essaouira in Morocco^59^, Haryana in India^60^, South Africa, and Malaysia^61^, but lower than that in Tunisia^62^, and Nanganur and Mothkur in southern India^63^. Given the importance of nitrate groundwater pollution for the safety of regional drinking water, timely monitoring is essential for minimizing the risk to human health.

## Conclusions

In conclusion, the groundwater on Hainan Island is mainly weakly alkaline freshwater, characterized as HCO_3_–Cl–Na and HCO_3_–Cl–Na–Ca. The chemical characteristics of groundwater are mainly affected by water–rock interactions, followed by cation alternating adsorption, and human activity. The WQI of 60.32% water sample points is less than 50, and the %Na of 85.71% is less than 60. The overall water quality is good, which is more suitable for drinking and irrigation, although the water quality is different in space. Compared with other areas, the water quality in the western part of the island is poor. Nevertheless, compared with other coastal areas, the average EC value of these samples is only 444.82, which is lower overall and more suitable for irrigation. The nitrate concentration range is 0–226.26 mg/L, and the nitrate pollution level is medium compared with areas of mainland China and other parts of the world. However, the non-carcinogenic risk of nitrate to infants is 36.51% higher than the acceptable value 1, which should be paid attention to. An appropriate level of development and management of water resources is essential, which must enable social development while maintaining use within the environmental carrying capacity. Simultaneously, the utilization efficiency of water resources needs to be improved by raising awareness of water quality and sustainability issues.

## Supplementary Information

Supplementary Information.

## Data Availability

The datasets used and/or analyzed during the current study are available from the corresponding author on request.
