# Supplementary material for: Assessment of groundwater hydrochemistry, water quality, and health risk in Hainan Island, China
Source: Sci Rep. 2023 Jul 26;13:12104. doi: 10.1038/s41598-023-36621-3 (PMC10372016; doi:10.1038/s41598-023-36621-3)
Supplement: Supplementary file 1 — Supplementary Information. [file 41598_2023_36621_MOESM1_ESM.docx]

**Supplementary Material**

**Assessment of groundwater hydrochemistry, water quality, and health risk in Hainan Island, China**

Qingqin Hou^a,1^, Yujie Pan^b,1^, Min Zeng^d^, Simiao Wang^c^, Huanhuan Shi^d^, Changsheng Huang^e^, Hongxia Peng^a,*^

^a^ School of Geography and Information Engineering, China University of Geosciences, Wuhan 430074, China

^b^ College of Environmental Sciences and Engineering, Peking University, Beijing, 100000, China

^c^ School of Mechanical Engineering and Automation, Northeastern University, Liaoning, 110819, China

^d^ School of Environmental Studies, China University of Geosciences, Wuhan 430074, China

^e^ Wuhan Center of Geological Survey of China Geological Survey, Wuhan, 430000, China

^*^ Corresponding author:

Hongxia Peng,

School of Geography and Information Engineering, China University of Geosciences, No. 68, Jincheng Street, East Lake New Technology Development Zone, Wuhan, Hubei Province 430078, P. R. China.

Email: [penghx@cug.edu.cn](mailto:penghx@cug.edu.cn)

Telephone: +8613163248181

^1^Qingqin Hou and Yujie Pan contributed equally to this work

**
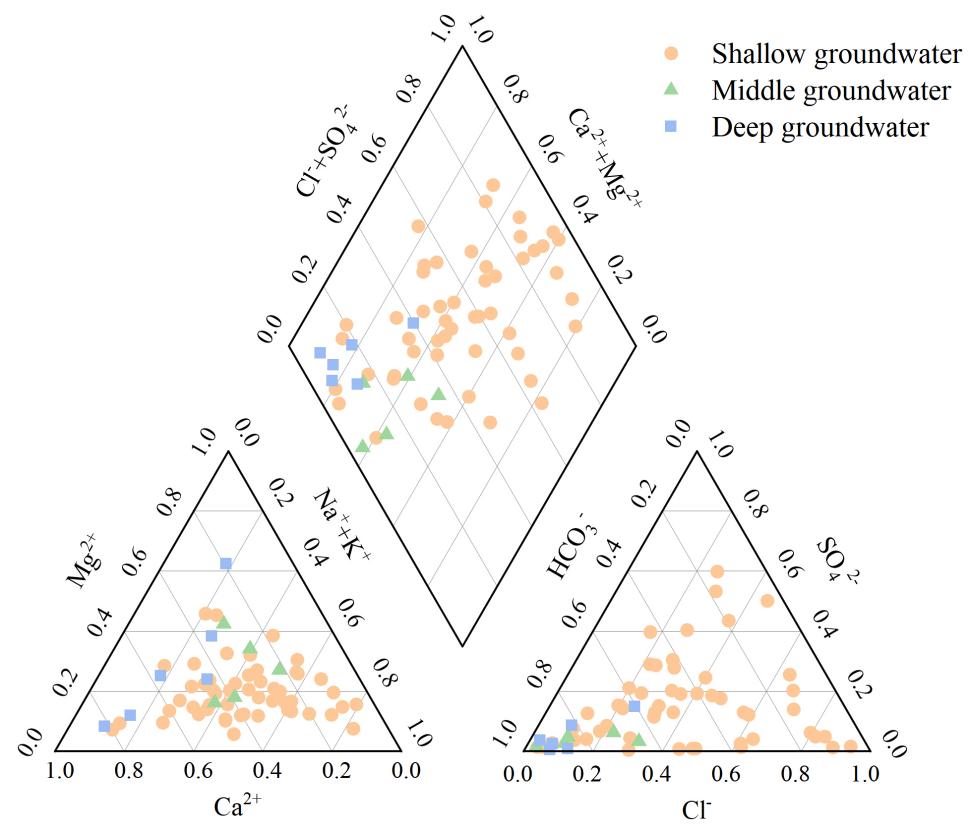
**

**Fig. S1** Piper diagrams of groundwater chemistry for Hainan Island, China.


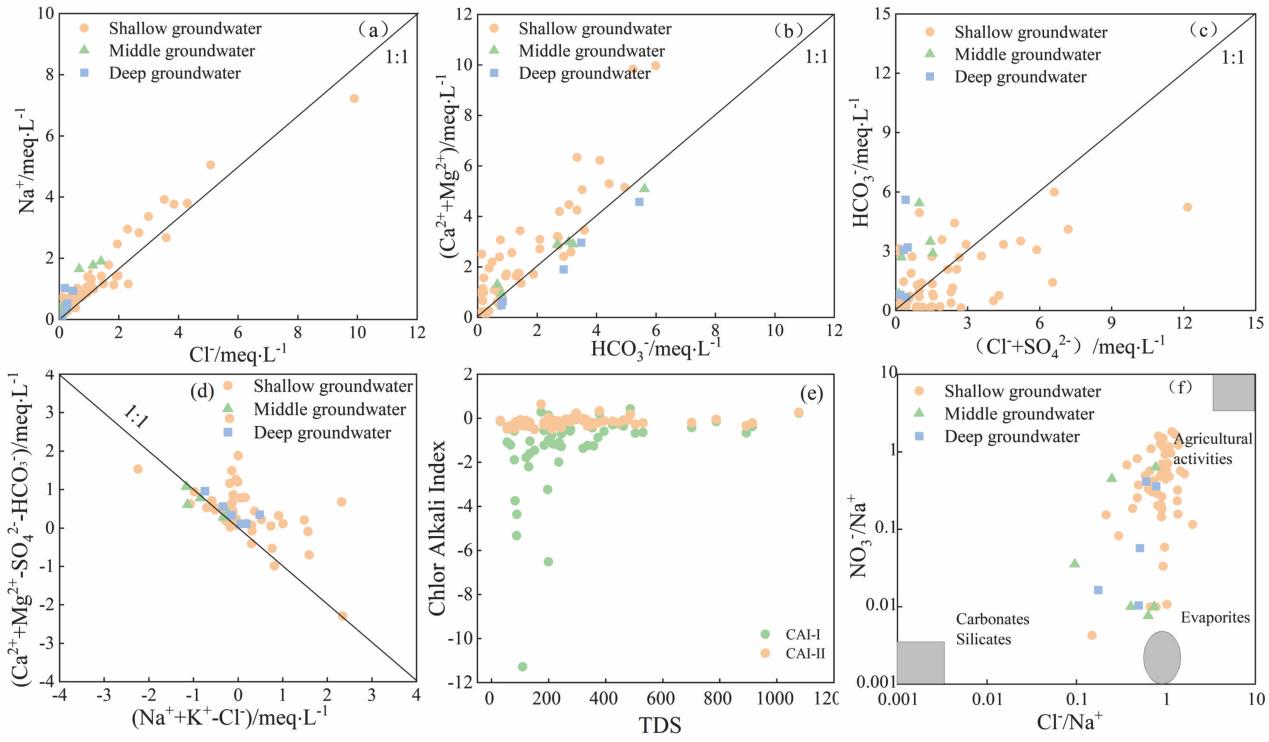


**Fig. S2** Relationship between hydro-chemical components (a) Na^+^ versus Cl^-^; (b) Ca^2+^ + Mg^2+^ versus HCO_3_^-^; (c) Cl^-^ + SO_4_^2-^ versus HCO_3_^-^; (d) Mg^2+^ + Ca^2+^－SO_4_^2^－HCO_3_^-^ versus Na^+^ + K^+^－Cl; (e) CAⅠ-Ⅰ and CAⅠ-Ⅱ indicating cation exchange; (f) Cl^-^/Na^+^ versus NO_3_^-^/Na^+^.


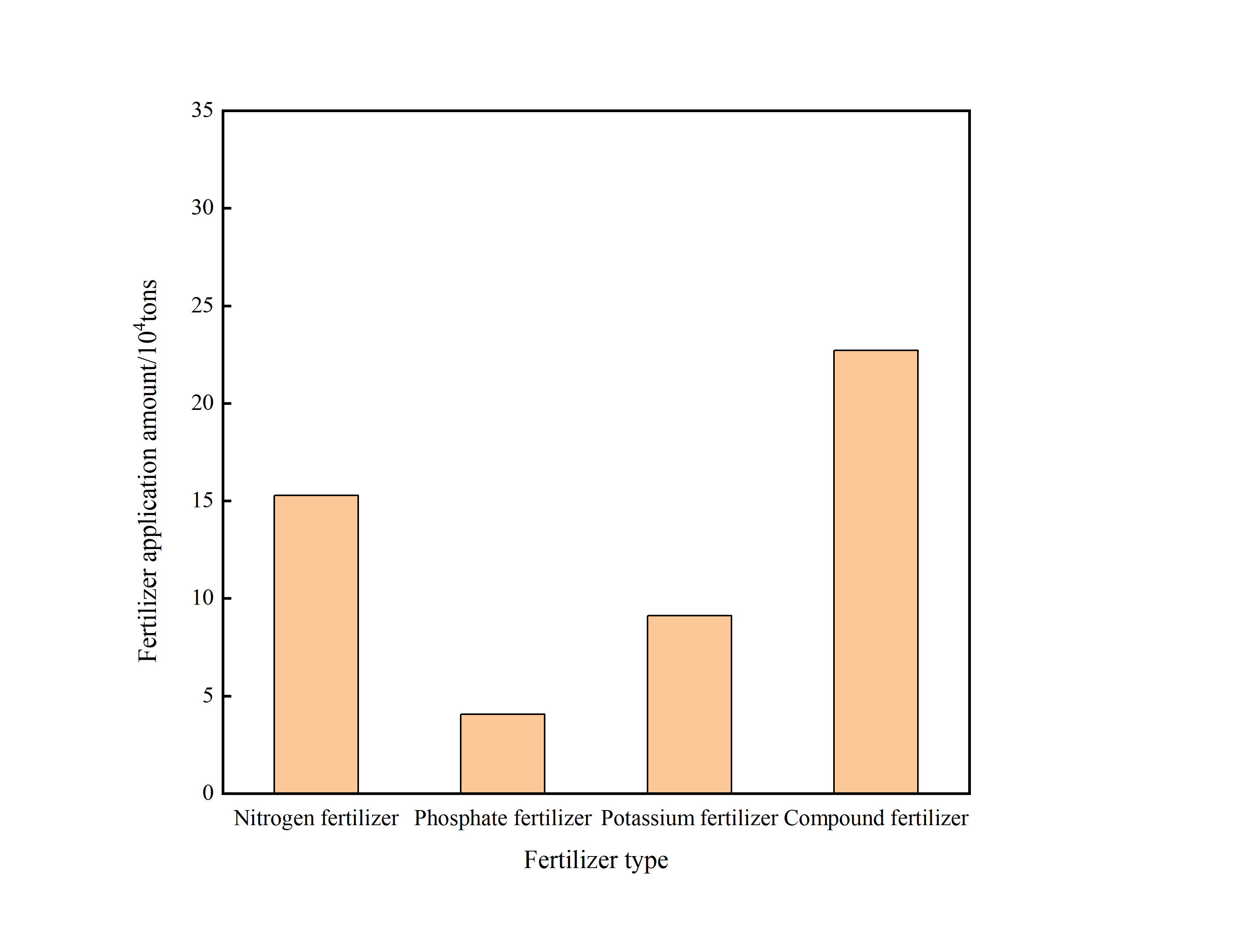


**Fig. S3** Fertilizer application amount in Hainan Island. The data from China Statistical Yearbook (http://www.stats.gov.cn/sj/ndsj).


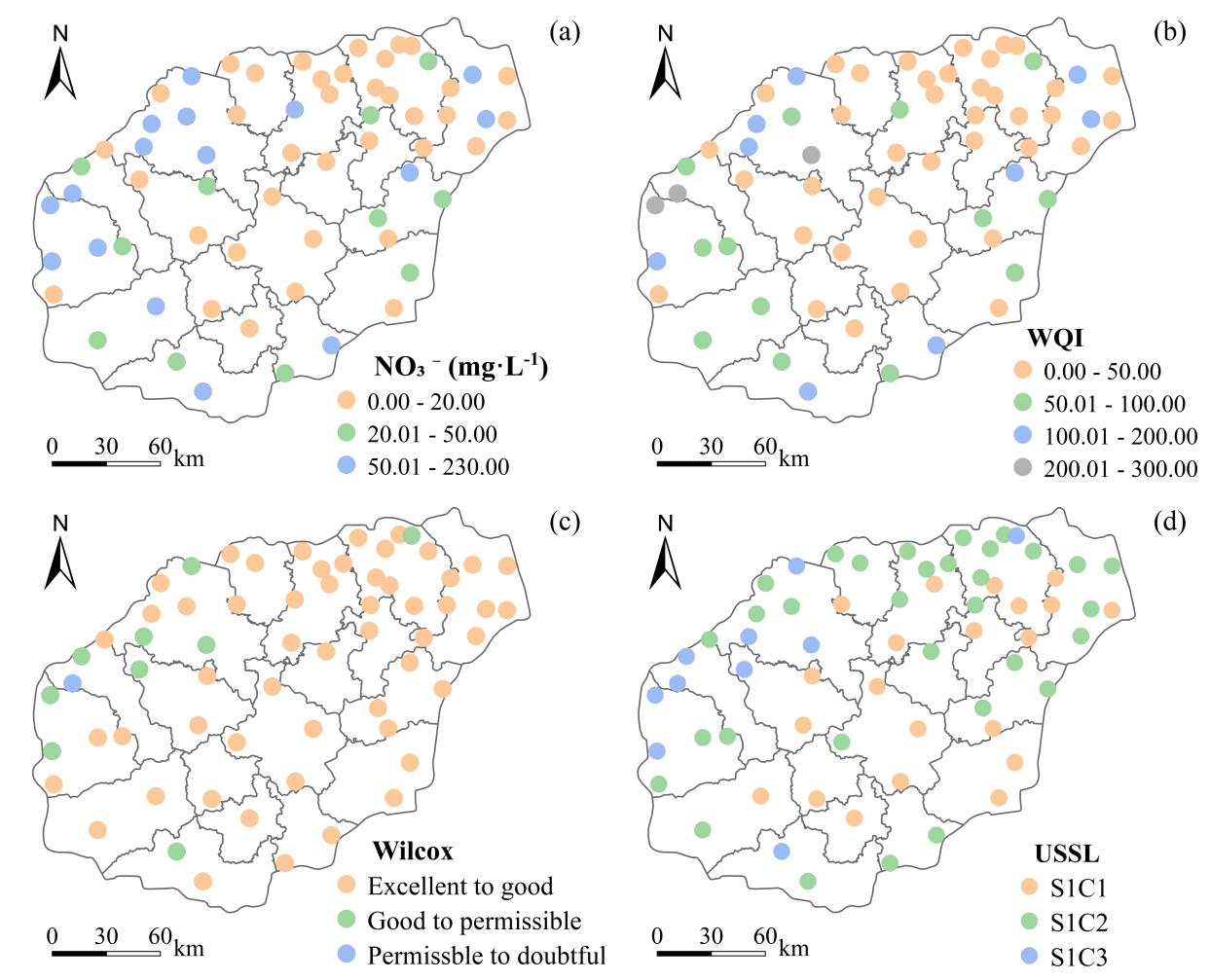


**Fig. S4** (a) Spatial distribution of nitrates; (b) spatial distribution of water quality index (WQI) values; (c) spatial distribution of the Wilcox test values; (d) spatial distribution of the USSL. The map was created using ArcGIS 10.8 (https://www.esri.com/software/ArcGIS).


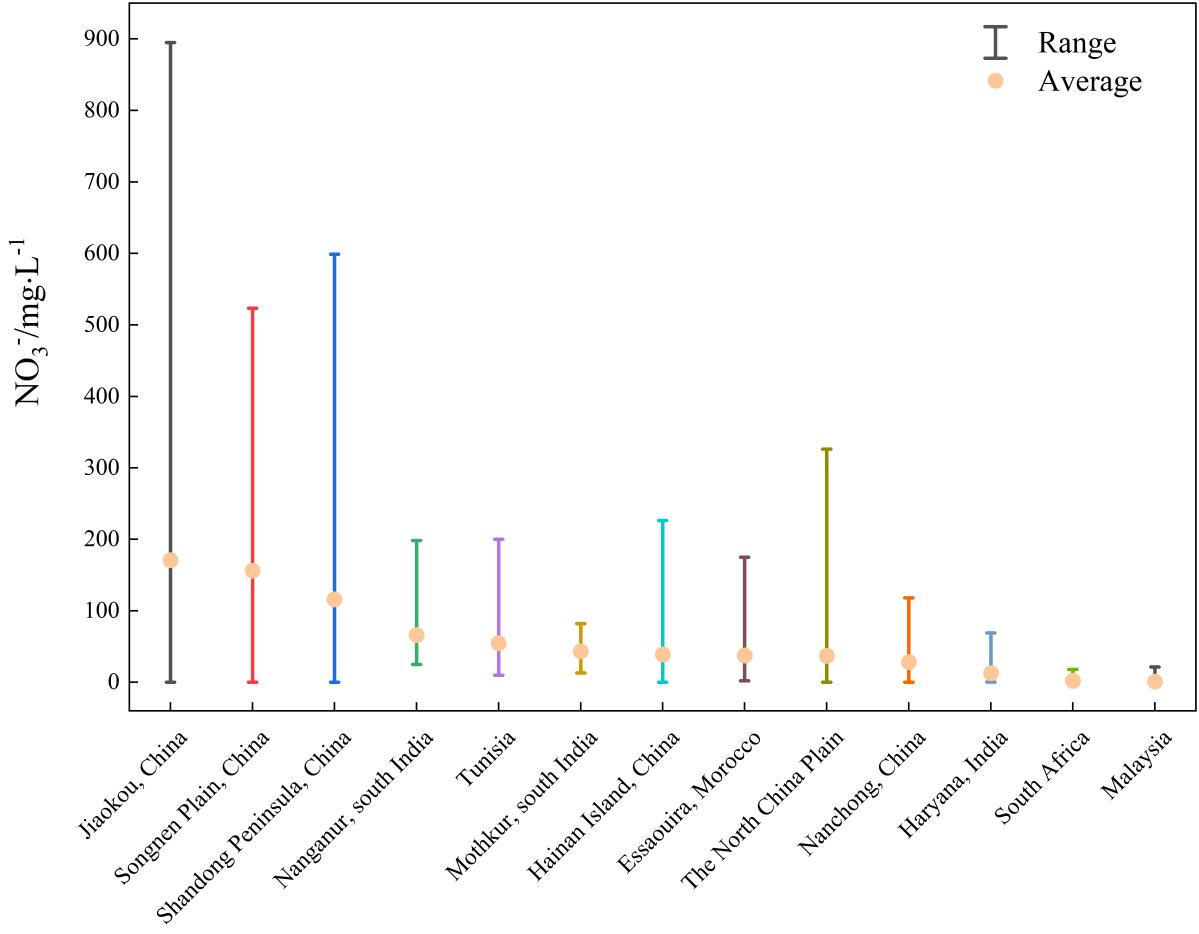


**Fig. S5** Comparison of nitrate concentrations between Hainan Island and other countries.

**Table S1** Relative weights and effective weights of water chemical parameters.

| Parameters | Permissible values  (SGQC, 2017) | Weight (w_i_) | Relative weight (W_i_) | Ew_i_ (%) | | |
| --- | --- | --- | --- | --- | --- | --- |
|  | | | | Min | Max | Mean |
| Na^+^ | 200 mg/L | 3 | 0.1154 | 0.98 | 10.10 | 3.28 |
| Cl^-^ | 250 mg/L | 4 | 0.1538 | 0.40 | 14.79 | 4.28 |
| SO_4_^2-^ | 250 mg/L | 4 | 0.1538 | 0.04 | 28.64 | 3.24 |
| NO_3_^-^ | 20 mg/L | 5 | 0.1923 | 0.00 | 87.18 | 43.67 |
| TH | 450 mg/L | 3 | 0.1154 | 0.78 | 24.48 | 6.94 |
| TDS | 1000 mg/L | 4 | 0.1538 | 2.95 | 19.35 | 8.85 |
| pH | 6.5–8.5 | 3 | 0.1154 | 3.77 | 78.72 | 29.73 |

**Table S2** Parameters for computing the human health risks associated with nitrate.

| Parameters | Infants | Children | Teenagers | Adults | References |
| --- | --- | --- | --- | --- | --- |
| RfD (mg⋅kg^−1^⋅d^−1^) | 1.6 | 1.6 | 1.6 | 1.6 | 33,34 |
| IR (L⋅d^−1^) | 0.57 | 1.03 | 1.27 | 1.4 | 35,36 |
| BW (kg) | 7.55 | 16.13 | 43.13 | 54 | 35,36 |
| AT (d) | 365×ED | 365×ED | 365×ED | 365×ED | 34 |
| ED (a) | 1 | 6 | 12 | 30 | 34 |
| EF (d⋅a^−1^) | 365 | 365 | 365 | 365 | 34 |

**Table S3** Fitted distributions of contaminant and exposure variables.

| Parameters | Infants | Children | Teenagers | Adults | References |
| --- | --- | --- | --- | --- | --- |
| NO_3_^-^(mg/L) | LN (38.92, 51.52) | | | |  |
| IR (L⋅d^−1^) | N (0.57, 0.47) | N (1.03, 0.48) | N (1.27, 0.58) | N (1.4, 0.8) | 35,36 |
| BW (kg) | LN (7.55, 1.1) | LN (16.13, 1.95) | LN (43.13, 8.9) | LN (54, 9.7) | 35,36 |
